# Application of metabolomics in toxicity evaluation of traditional Chinese medicines

**DOI:** 10.1186/s13020-018-0218-5

**Published:** 2018-12-04

**Authors:** Li Duan, Long Guo, Lei Wang, Qiang Yin, Chen-Meng Zhang, Yu-Guang Zheng, E.-Hu Liu

**Affiliations:** 1 College of Chemistry and Material Science, Hebei Normal University, Shijiazhuang, 050024 China; 2 School of Pharmacy, Hebei University of Chinese Medicine, Shijiazhuang, 050200 China; 3 State Key Laboratory of Natural Medicines, China Pharmaceutical University, Nanjing, 210009 China; 4 Hebei Key Laboratory of Chinese Medicine Research on Cardio-cerebrovascular Disease, Hebei University of Chinese Medicine, Shijiazhuang, 050200 China; 5 Department of Management, Xinjiang Uygur Pharmaceutical Co., Ltd., Wulumuqi, 830001 China

**Keywords:** Traditional Chinese medicines, Toxicity, Metabolomics, Toxicity mechanisms

## Abstract

Traditional Chinese medicines (TCM) have a long history of use because of its potential complementary therapy and fewer adverse effects. However, the toxicity and safety issues of TCM have drawn considerable attention in the past two decades. Metabolomics is an “omics” approach that aims to comprehensively analyze all metabolites in biological samples. In agreement with the holistic concept of TCM, metabolomics has shown great potential in efficacy and toxicity evaluation of TCM. Recently, a large amount of metabolomic researches have been devoted to exploring the mechanism of toxicity induced by TCM, such as hepatotoxicity, nephrotoxicity, and cardiotoxicity. In this paper, the application of metabolomics in toxicity evaluation of bioactive compounds, TCM extracts and TCM prescriptions are reviewed, and the potential problems and further perspectives for application of metabolomics in toxicological studies are also discussed.

## Background

Traditional Chinese medicines (TCM) have been used for the treatment of a variety of diseases for thousands of years in China since they are relatively inexpensive, widely available and have reliable therapeutic efficacy [1–3]. Accompanying with hot discussions on development of multidrug therapy for multi-gene diseases, TCM are receiving increasing attention worldwide because it is well accepted that TCM exert their curative effects via multiple components on multiple targets in clinic [4–6].

Many people believe that TCM are safe because they derive from natural origin. However, this belief has been greatly challenged in recent years. In fact, the toxicity and safety issues of TCM has aroused increasing concern to international community, such as identification of plant materials, preparation method, and the potential to interact with other herbal medicines and conventional drugs [7–10]. Moreover, the traditional safety assessment methods may not accurately assess safety knowledge of TCM due to the complexity of its constituents and action mechanisms.

Systems biology is a biology-based interdisciplinary field of study that focuses on complex interactions within biological systems, using a holistic approach to biological research [11]. Indeed, the holistic properties of systems biology are in agreement with TCM theory in nature [12, 13]. The omics approaches, such as genomics, transcriptomics, proteomics and metabolomics, have greatly facilitated the systematic study of complex systems, especially TCM and herbal medicines [14–16].

Metabolomics, first put forward by professor Nicholson in 1999 [17], is defined as systematically qualitative and quantitative analysis of metabolites in a given organism or biological sample. It allows the quantitative measurement of large numbers of low-molecular-weight (< 1 kDa) endogenous metabolites, including lipids, amino acids, peptides, nucleic acids, organic acids, vitamins, and carbohydrates, which play important roles in biological systems and represent attractive candidates to understand phenotypes [18–20]. Metabolomics is suitable for observing abnormal changes of endogenous metabolites before the appearance of physiological or pathological damages. As a systemic approach, metabolomics adopts a “top-down” strategy to reflect the function of organisms from terminal symptoms of metabolic network and understand metabolic changes of a complete system caused by interventions in a holistic context [21].

Recently, metabolomics has been widely applied to the modern researches of TCM including theory of TCM, syndrome, efficacy and toxicity since the metabolome represents the physiological or pathological status of organisms [22–25]. It was deemed that metabolomic analysis is an efficacious and noninvasive method to evaluate toxicity of TCM and explore toxicity mechanisms through correlations of physiological changes and metabolic changes [26, 27]. In this review, we summarized the metabolomics analytical techniques widely used in the study of TCM, and focusing on the application of metabolomics in the toxicological evaluation of TCM.

### Metabolomic technology and data analysis

Modern metabolomic technologies allow for qualitative and quantitative measurement of a vast number of metabolites in complex biological systems. The main analytical techniques in metabolomics, which have widespread applications in the assessments of efficacy and toxicology of TCM, are proton nuclear magnetic resonance spectroscopy (^1^H NMR) and mass spectrometry (MS) [28].

^1^H NMR is a non-destructive technique, which provides high-throughput and automated analysis of crude extracts, and quantitatively detects different metabolites in different groups, as well as offers structural information [29]. The advantages of ^1^H NMR in metabolomic analysis include simple and nondestructive sample preparation, fast analysis rate, and non-selective judgment. However, ^1^H NMR fails to obtain valid data when the concentrations of metabolites in complex sample are quite low [30]. Therefore, in most cases, MS is preferred in metabolomic analysis because of its advantages of unparalleled sensitivity, high resolution and structural specificity [31]. In practical applications, MS requires combining with different separation techniques such as gas chromatography (GC–MS), liquid chromatography (LC–MS), capillary electrophoresis (CE–MS) and ultra-performance liquid chromatography (UPLC-MS) for a pre-separation. GC–MS is particularly suitable for the detection of thermally stable volatile metabolites. Hence, the application range of GC–MS is limited as most non-volatile metabolites cannot be analyzed directly [32]. Compared to GC–MS, the utilization of LC–MS is more frequent in metabolomic analysis, LC can isolate different kinds of metabolites in a complex system and MS can provide structural information to help to identify metabolites. LC–MS can provide more details of submerged portions than ^1^H NMR, and can detected molecules with different proper polarity [33]. The ability of LC–MS to analyze various kinds of metabolites depends on the ionization source and the chromatographic method that is used to separate a complex mixture of analytes. Nowadays, two-dimensional LC method has been successfully applied in metabolomic analysis of TCM and due to its enhanced selectivity, peak capacity and high resolution compared with one-dimensional LC [34]. Normally, the selection of metabolomic technology depends on the research purpose and the properties of samples. In fact, due to the large number and the wide concentration range of metabolites, and the complexity of TCM, integrated metabolomic approaches have been frequently used to provide sensitive, accurate and reliable results [35].

Sample preparation, including its source, storage and extraction, has significant effects on the results of metabolomic analysis. Plasma, serum, urine and tissue are usually biological samples in metabolomic analysis [36]. To decrease the changes of potential metabolites in metabolomic samples, biological samples usually can be restored in − 80 °C. For ^1^H NMR analysis, the change of pH and ionic strength caused by the change of the chemical shift is the primary problem, and the addition of pH buffer during the sample extraction can solve the problem [37]. Compared with ^1^H NMR, the samples extraction for MS-based metabolomics are more complicated. For LC–MS analysis, biological samples are complex and contain various endogenous and exogenous acidic, basic, and neutral compounds with high polarity. The samples usually require to be centrifuged and diluted with deionized water before metabolomic analysis [38]. For GC–MS analysis, most potential biomarkers in biological samples are high polar and nonvolatile, thus the samples must be derivatized before analysis [39].

**Fig. 1:**
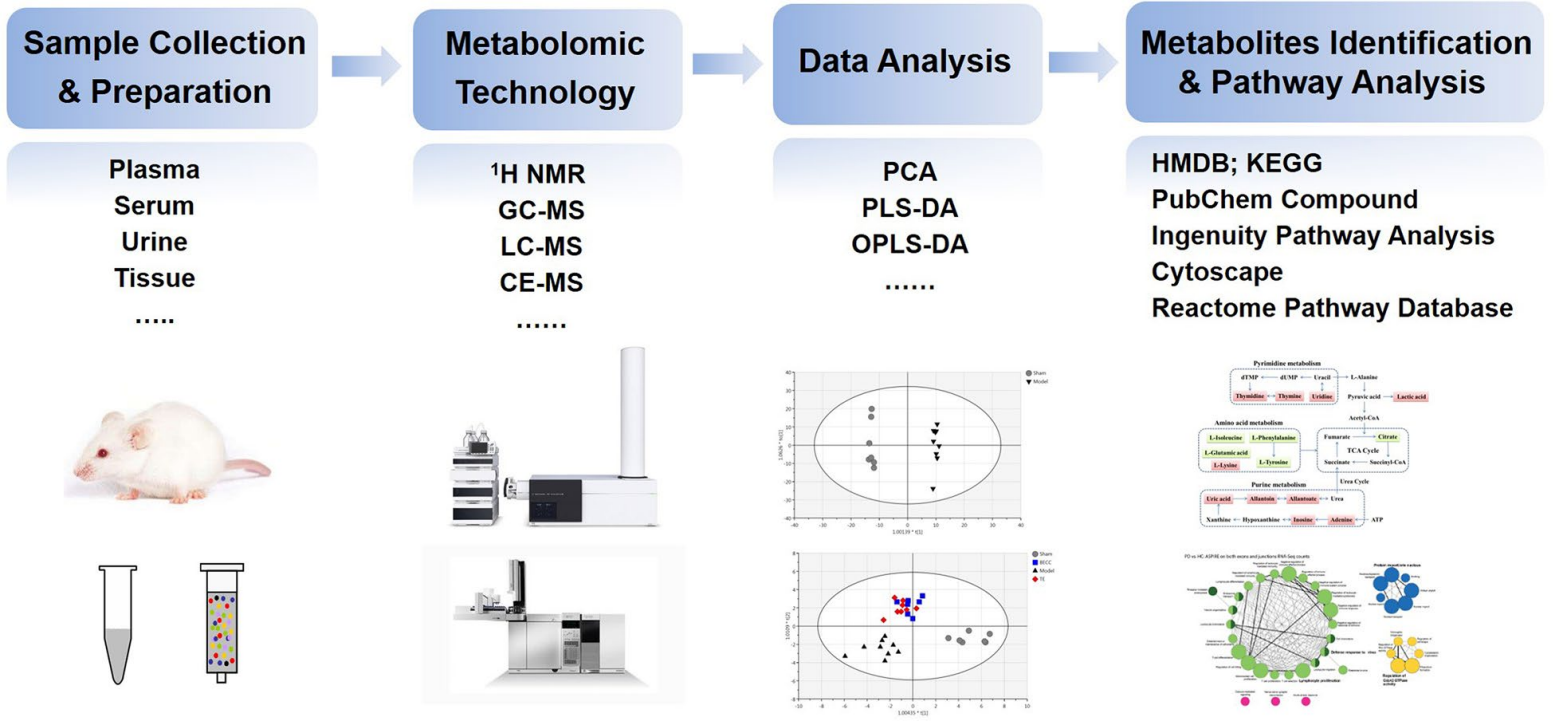
The flowchart of typical metabolomic analysis

Data analysis are crucial since the data matrix generated in metabolomic study is generally large and complex. Data preprocessing is the first step of metabolomic data analysis. The main objective of data preprocessing is to transform the data in such way that the samples in the dataset are more comparable in order to ease and improve the data analysis [40]. ^1^H NMR data preprocessing usually includes baseline correction, alignment, binning, normalization and scaling [41]. For MS data preprocessing, many softwares such as MetAlign, MZmine and XCMS have been developed to process raw data [42]. Multivariate statistical methods are professional approaches for analyzing and maximizing information retrieval from complex metabolomic data. The multivariate statistical methods can be classified into two groups, namely unsupervised methods and supervised methods. Unsupervised methods mainly include principal component analysis (PCA), hierarchical cluster analysis (HCA), K-means and statistical total correlation spectroscopy. PCA can summarize the information in an experimental data set using a small number of orthogonal latent variables obtained by searching the direction of maximum variance in the data set. However, PCA does not always extract hidden information that explains system behavior. Supervised methods, such as partial least squares discriminant analysis (PLS-DA), orthogonal partial least squares discriminant analysis (OPLS-DA), quadratic discriminant analysis and linear discriminant analysis can reveal the most important factors of variability characterizing the metabolomic datasets [43]. The commonly used softwares for metabolomic multivariate statistical analysis are Shimadzu Class-VP software and SIMCA-P software. The identification of metabolites and the pathway analysis of metabolites are also essential components of metabolomic data analysis. The updating commercial software is crucial for identifying potential metabolites, while accurate mass, isotopic pattern, fragments information, and available biochemical databases are also necessary. Presently, a number of metabolites databases such as Human Metabolome Database (HMDB), Kyoto Encyclopedia of Genes and Genomes (KEGG), Biochemical Genetic and Genomic (BiGG), ChemSpider and PubChem Compound, are emerging and have been applied in the identification of metabolites and biomarkers. For metabolic pathway analysis, KEGG, Ingenuity Pathway Analysis, Cytoscape and Reactome Pathway Database are commonly used databases and softwares. The flowchart of typical metabolomic experiment including sample preparation, metabolomic technology, data analysis and pathway analysis is shown in Fig. 1.

### Metabolomics in toxicity evaluation of TCM

Metabolomic analysis is an effectively and noninvasive method for evaluating toxicology of TCM and exploring toxicity mechanisms through correlations of physiological changes and metabolic changes. The metabolomic researches on hepatotoxicity, nephrotoxicity, cardiotoxicity and other toxicity induced by bioactive compounds, TCM extracts and TCM prescriptions were summarized in Tables 1, 2, 3 and 4, respectively.

**Table 1:** The applications of metabolomics in hepatotoxicity evaluation of TCM

TCM	Toxicity	Animals	Samples	Metabolomic technology	Data analysis	Metabolic pathways	References
Triptolide	Hepatotoxicity	Mice	Serum, liver	LC–MS	PCA, PLS-DA	Glutathione metabolism, tricarboxylic acid cycle, purine metabolism, glycerophospholipid metabolism, taurine and hypotaurine metabolism, pantothenate and coenzyme A biosynthesis, pyrimidine metabolism, amino acid metabolism	[45, 46]
Dioscorea bulbifera Rhizome	Hepatotoxicity	Rats	Plasma, urine, feces	GC–MS	OPLS-DA	Amino acid metabolism, bile acid metabolism, purine metabolism, pyrimidine metabolism, lipid metabolism, energy metabolism	[49]
		Rats	Urine	^1^H NMR	PLS-DA	Amino acid metabolism, fatty acid metabolism, energy metabolism	[50]
Xanthii Fructus	Hepatotoxicity	Rats	Urine	LC–MS	PCA, PLS-DA, OPLS-DA	Mitochondrial inability, fatty acid metabolism, amino acids metabolism	[52, 53]
Polygoni multiflori radix	Chronic hepatotoxicity	Rats	Serum	GC–MS	PCA, PLS-DA	Amino acid metabolism, fatty acid metabolism, oxidative injury	[55]
Realgar	Hepatotoxicity	Rats	Urine	LC–MS, ^1^H NMR	PCA, OPLS-DA	Citric acid cycle, tryptophan metabolism, porphyrin metabolism	[57]
	Sub-chronic hepatotoxicity	Mice	Plasma, urine	^1^H NMR	PCA	Energy metabolism, amino acids metabolism, gut bacteria metabolism	[58]

**Table 2:** The applications of metabolomics in nephrotoxicity evaluation of TCM

TCM	Toxicity	Animals	Samples	Metabolomic technology	Data analysis	Metabolic pathways	References
Aristolochic acid	Nephrotoxicity	Rats	Urine	GC–MS	PCA, PLS-DA	Energy metabolism, gut microbiota, purine metabolism	[60]
		Rats	Urine	LC–MS	OPLS-DA	Tricarboxylic acid cycle, gut microflora metabolism, amino acid metabolism, purine metabolism, bile acid biosynthesis	[61]
Strychni Semen	Nephrotoxicity	Rats	Serum, urine	^1^H NMR	OPLS-DA	Glycolysis, lipid and amino acid metabolism	[64]
Arisaematis Rhizoma	Nephrotoxicity	Rats	Serum, urine	^1^H NMR	PLS-DA	Glycolysis–gluconeogenesis, tricarboxylic acid cycle, fatty acid metabolism, gut microflora metabolism	[66]
Pharbitidis Semen	Nephrotoxicity	Rats	Serum, kidney	LC–MS	PCA	Phospholipids metabolism, amino acid metabolism, sphingolipids biosynthesis and metabolism	[68]
		Rats	Urine	LC–MS	PCA	Amino acid metabolism, citric acid cycle, bile acid metabolism	[69]
Alismatis Rhizoma	Chronic nephrotoxicity	Rats	Urine	LC–MS	PCA	Amino acid metabolism, Purine metabolism, bile acid metabolism, sphingolipids metabolism	[71]

**Table 3:** The applications of metabolomics in cardiotoxicity evaluation of TCM

TCM	Toxicity	Animals	Samples	Metabolomic technology	Data analysis	Metabolic pathways	References
Periplocin	Cardiotoxicity	Neonatal rats	Cardiomyocytes	LC–MS	PCA, PLS-DA, Support Vector Machine	Amino acid metabolism, energy metabolism, sphingolipid metabolism	[73]
*Aconitum* alkaloids	Cardiotoxicity	Rats	Plasma	^1^H NMR, GC–MS	PCA	Energy metabolism, fatty acid metabolism, amino acid metabolism, purine metabolism	[74]
Aconiti kusnezoffii Radix	Cardiotoxicity	Rats	Urine	LC–MS	PCA, PLS-DA, OPLS-DA	Pentose and glucuronate interconversions, tryptophan metabolism, amino sugar and nucleotide sugar metabolism, taurine and hypotaurine metabolism, ascorbate and aldarate metabolism, fructose and mannose metabolism, starch and sucrose metabolism	[77]
Aconiti Radix	Cardiotoxicity	Rats	Urine	LC–MS	PCA, PLS-DA, OPLS-DA	pentose and glucuronate interconversions, amino acid metabolism, starch and sucrose metabolism, amino sugar and nucleotide sugar metabolism, purine metabolism, tryptophan metabolism, taurine and hypotaurine metabolism, fructose and mannose metabolism, fatty acid metabolism	[78, 79]
Aconiti Lateralis Radix Praeparata	Cardiotoxicity	Rats	Plasma	LC–MS	PCA, PLS-DA, OPLS-DA	Sphingolipid metabolism, aminoacyl-tRNA biosynthesis, tryptophan metabolism	[80]
		Mices	Heart	LC–MS	PCA, PLS-DA	Phospholipid metabolism, Sphingolipid metabolism, saturated fatty acid oxidation, unsaturated fatty acid peroxidation	[81]
Pinelliae Rhizoma	Cardiotoxicity	Rats	Serum	LC–MS	PCA, PLS-DA	Phospholipid metabolism, amino acid metabolism, carnitine metabolism	[83]

**Table 4:** The applications of metabolomics in other toxicity evaluation of TCM

TCM	Toxicity	Animals	Samples	Metabolomic technology	Data analysis	Metabolic pathways	References
Triptolide	Reproduction toxicity	Mice	Serum, testis	GC–MS	PLS-DA	Lipid metabolism, energy metabolism	[85]
Cinnabar	Neurotoxicity	Rats	Brain	^1^H NMR	PCA	Glutamate metabolism, membrane disruption, energy metabolism, oxidative injury	[87]
Kunsui Radix	Inflammation, irritation to the skin, tumor-promotion	Rats	Urine	^1^H NMR	PCA	Tricarboxylic acid cycle, anaerobic glycolysis, amino acids metabolism	[89]
Coptidis Rhizome	Diarrhea	Rats	Serum, urine	^1^H NMR, GC–MS	PCA, OPLS-DA	Gut microflora metabolism, energy metabolism, amino acids metabolism	[90]
Niuhuang Jiedu Tablet	Hepatotoxicity, nephrotoxicity	Rats	Urine	^1^H NMR	PCA, PLS-DA	Energy metabolism, choline metabolism, amino acid metabolism, gut flora disorder	[92]
Zhusha Anshen Wan	Hepatotoxicity, nephrotoxicity	Rats	Serum, urine	^1^H NMR	PLS-DA	Energy metabolism, lipid metabolism, choline metabolism	[93]
Shuanghuanglian injection	Hemolytic anemia	Dogs	Serum	^1^H NMR	PCA	Cell membranes metabolism, lipid metabolism, energy metabolism, amino acid metabolism, gut microflora metabolism	[95]

### Metabolomics in hepatotoxicity evaluation of TCM

Metabolomics is a useful tool to evaluate toxicity and identify toxicological biomarkers of bioactive compounds from TCM. Triptolide, a bioactive diterpenoid compound isolated from *Tripterygium wilfordii*, exhibits diverse biological activities such as anti-inflammatory, immune-modulatory and anti-proliferative activities [44]. However, the further clinical research and application of triptolide is confined by its severe toxicity on the liver, kidney and reproductive systems [45]. Zhao et al. developed a LC–MS based metabolomic method to investigate the hepatotoxicity of triptolide in mice. Mice were administered triptolide by gavage to establish the acute liver injury model. Metabolomic results showed that a total of thirty metabolites were significantly changed by triptolide treatment and the abundance of twenty-nine metabolites was correlated with toxicity. Pathway analysis indicated that the mechanism of triptolide induced hepatotoxicity was related to alterations in multiple metabolic pathways, including glutathione metabolism, tricarboxylic acid cycle, purine metabolism, glycerophospholipid metabolism, taurine and hypotaurine metabolism, pantothenate and coenzyme A biosynthesis, pyrimidine metabolism and amino acid metabolism [46]. Recently, another LC–MS based metabolomic approach was developed to discover hepatotoxic and nephrotoxic potential biomarkers of triptolide. The metabolic profiles of liver, kidney and plasma were characterized by HPLC Q/TOF MS. The metabolite profiles of the liver, kidney and plasma of toxic and therapeutically dosed mice showed significant differences. Two toxic markers, mono-hydroxylated metabolite of triptolide, tri-hydroxylated and dehydrogenated metabolite of triptolide, were detected both in mice plasma and human liver microsomes following incubation with triptolide. The two metabolites could be potential diagnosis markers for hepatotoxicity and nephrotoxicity induced by triptolide [45]. The metabolomic analysis could provide an integral understanding of the mechanism of the hepatotoxicity, and it may be useful for further prediction and diagnosis of liver injury during clinical use of triptolide.

Compared with the limited applications in toxicity evaluation of bioactive compounds, metabolomics was widely applied to toxicity evaluation of the TCM extracts. Dioscorea bulbifera Rhizome, the dried root of *Dioscorea bulbifera* L., has been known to have many bioactivities such as anti-tumor, anti-bacterial, anti-feedant, anti-fungal and anti-salmonella [47]. However, experimental studies and clinical reports indicated that Dioscorea bulbifera Rhizome could cause toxicity, particularly in the liver [48]. A multisample integrated metabolomic strategy was employed to precisely describe the status and mechanism of hepatotoxicity induced by Dioscorea bulbifera Rhizome. Comparison of metabolomic profiles of rat plasma, urine, and feces by GC–MS, a total of fifty-five metabolites distributed in 33 metabolic pathways were identified. Correlation network analysis revealed that the hub metabolites of hepatotoxicity were mainly associated with amino acid metabolism, bile acid metabolism, purine metabolism, pyrimidine metabolism, lipid metabolism and energy metabolism [49]. In another study, liver toxicity induced by Dioscorea bulbifera Rhizome was investigated by ^1^H NMR. The metabolomic results revealed that the levels of taurine, creatine, betaine, dimethylglycine, acetate, glycine were elevated, whereas, the levels of succinate, 2-oxoglutarate, citrate, hippurate and urea were reduced. With molecular function analysis of these changed metabolites, the hepatotoxicity of Dioscorea bulbifera Rhizome was involved in hepatic mitochondrial injury [50].

Xanthii Fructus is the mature fruit with involucres of *Xanthium sibirium* Patr. and widely used for the treatment of sinusitis, headache, rheumatism, and skin itching [51]. Xue et al. performed an integrated metabolomic study using ^1^H NMR combined with multivariate statistical analysis to elucidate the hepatotoxicity of Xanthii Fructus. When rats were treated with Xanthii Fructus at 30.0 g/kg, the hepatotoxicity was reflected in the changes observed in serum biochemical profiles and by the histopathological examination of the liver. The results demonstrated that atractyloside, carboxyatractyloside and 40-desulphate-atractyloside were the major hepatotoxicity constituents in Xanthii Fructus. Moreover, the hepatotoxicity of Xanthii Fructus mainly associated with mitochondrial inability, fatty acid metabolism, and some amino acids metabolism [52]. The urinary metabolic perturbations associated with toxicity induced by Xanthii Fructus were also studied using UPLC–MS. The results showed that the metabolic characters in Xanthii Fructus treated rats were perturbed in a dose dependent manner and ten metabolites including 6-hydroxy-5-methoxyindole glucuronide/5-hydroxy-6-methoxyindole glucuronide, 4,6-dihydroxyquinoline, 3-methyldioxyindole, phenylalanine, indoxyl sulfate, hippuric acid, uridine, l-phenylalanyl-l-hydroxyproline, sebacic acid, and arachidonic acid were preliminarily identified as potential toxicity biomarkers [53].

Polygoni Multiflori Radix, the dried root of *Polygonum multiflorum* Thunb, is commonly used to prevent or treat non-alcoholic fatty liver disease, hyperlipidemia or related hepatic diseases in clinic. Currently, several clinical cases associated with hepatotoxicity of Polygoni Multiflori Radix including toxic hepatitis and acute hepatitis have been reported [54]. Xia et al. used an untargeted metabolomic strategy to investigate the chronic hepatotoxicity induced by Polygoni Multiflori Radix in rats. Ten potential endogenous metabolites including glycine, 13-eicosenoic acid, lactic acid, octadecanoic acid, proline, 2-furoic acid, cholesterol, alanine, docosahexaenoic acid, and lysine were identified. The ten potential biomarkers were involved in three metabolic pathways, amino acid metabolism, fatty acid metabolism and oxidative injury. The results indicated that Polygoni Multiflori Radix-induced liver damage is dosage dependent and disruption in amino acid and energy metabolism might lead to subsequent oxidative damage in the liver of rats [55].

Realgar, an ore crystal containing more than 90% tetra-arsenic tetrasulfide, has been used for the treatment of carbuncles, boils, insect-and snake-bites, intestinal parasitosis, convulsive epilepsy and psoriasis [56]. As an arsenical, realgar is known as a poison and paradoxically as a therapeutic agent. Using a combined LC–MS and ^1^H NMR based metabolomic approach, Huang et al. investigated the hepatotoxicity induced by realgar in rats. Thirty-six potential biomarkers were discovered, and these metabolites were distributed in citric acid cycle, tryptophan metabolism, and porphyrin metabolism. Glycine and serine were proposed as key metabolites related to realgar-induced disturbance [57]. In another study, a ^1^H NMR-based metabolomic approach was employed to investigate the subchronic hepatotoxicity of realgar on mice. The change trends of metabolites in urine and plasma from mice subchronic exposed to realgar are similar to those acute exposed to realgar, which indicate the acute and sub-chronic toxic mechanisms of realgar are same. The disturbed metabolic pathways include energy metabolism, amino acids metabolism and gut bacteria metabolism [58].

### Metabolomics in nephrotoxicity evaluation of TCM

Aristolochic acid is a mixture of structural-related nitrophenanthrene carboxylic acid derivatives existed in *Aristolochia, Bragantia and Asarum* genus, such as Aristolochiae Fructus, Stephaniae tetrandrae Radix and Asari Ridix et Rhizoma [59]. Aristolochic acid is a toxicant that can cause a common and rapidly progressive interstitial nephropathy called aristolochic acid nephropathy. The pathophysiology and underlying mechanisms of the aristolochic acid nephropathy have been studied using metabolomic approach by different analysis methods. Hu et al. employed a GC–MS based metabolomic technique to analyze urinary metabolites in aristolochic acid treated rats. Eight metabolites were selected as potential metabolic biomarkers including methylsuccinic acid, nicotinamide, 3-hydroxyphenylacetic acid, citric acid, creatinine, uric acid, glycolic acid, and gluconic acid. The identified metabolites suggested that the pathways of energy metabolism, gut microbiota, and purine metabolism were associated with aristolochic acid induced nephrotoxicity [60]. In another LC–MS based urinary metabolomic study, the results suggested that the nephrotoxicity of aristolochic acid could be characterized via systemic disturbance of metabolic network including tricarboxylic acid cycle, gut microflora metabolism, amino acid metabolism, purine metabolism and bile acid biosynthesis, which were partly consistent with the results of GC–MS based metabolomic study [61].

Strychni Semen, the dried ripe seeds of *Strychnos nux*-*vomica* Linn., was commonly used to relieve rheumatism, induce analgesia, remove stasis, clear heat, and alleviate swelling in China [62]. However, the clinical applications of Strychni Semen is limited by its severe toxicity, especially nephrotoxicity. Fan et al. established a ^1^H NMR based metabolomic method to evaluate the toxicity induced by Strychni Semen. The results indicated that Strychni Semen induced disruptions in glycolysis, lipid and amino acid metabolism, and the toxic effects were aggravated in liver and kidney tissues as dosing time was prolonged [63]. A cell metabolomic strategy was also developed to investigate the nephrotoxicity of Strychni Semen. A total of 10 biomarkers and 24 related metabolic pathways were screened. The possible mechanisms of Strychni Semen nephrotoxicity might be cellular component disruption, oxidative damage, metabolic waste accumulation and the disturbance of energy and ion transport systems [64]. Metabolomics could be an efficient means to elucidate the mechanism of Strychni Semen-induced nephrotoxicity and might contribute to investigation of possible nephrotoxic mechanisms of other TCM.

Arisaematis Rhizoma, the dried rhizomes of *Arisaema erubescens* Schott, *Arisaema heterophyllum* BI. and *Arisaema amurense* Maxim., has been widely used due to its various effects including analgesic, sedative, stomachic, anticoagulant, antiemetic, anti-inflammatory and antitumor activities [65]. A ^1^H NMR based metabolomic approach complemented with serum chemistry and histopathology has been applied to investigate the nephrotoxicity of Arisaematis Rhizoma. The results indicated that thirteen metabolites in urine and six metabolites in serum were significantly altered, suggesting disturbances in energy metabolism, perturbation of the gut microflora environment, membrane damage, folate deficiency and injury of kidneys produced by Arisaematis Rhizoma [66].

Pharbitidis Semen, the dried mature seeds of *Pharbitis nil* (L.) Choisy or *Pharbitis purpurea* (L.) Voigt, is widely used for treatment of edema, simple obesity and lung fever in China and some east Asian countries. Several animal and clinical studies have reported the nephrotoxicity of Pharbitidis Semen [67]. Recently, a LC–MS based metabolomic approach was employed to delineate the comprehensive mechanism of nephrotoxicity induced by Pharbitidis Semen. The results indicated that certain metabolic pathways, such as lysophosphatidylcholines formation and sphingolipids cycle were accelerated [68]. Ma et al. performed another LC–MS based urinary metabolomics to investigate the nephrotoxicity induced by Pharbitidis Semen. The results indicated that ethanol extract of Pharbitidis Semen should be responsible for the nephrotoxicity and eight metabolites were identified. According to the identified metabolites, the underlying regulations of Pharbitidis Semen perturbed metabolic pathways were amino acid metabolism, citric acid cycle and bile acid metabolism [69].

Alismatis Rhizoma, the dried rhizome of *Alisma orientale* (Sam.) Juz., has been widely used as diuretic, antinephrolithic, hypolipidemic, antiatherosclerotic, antidiabetic and anti-inflammatory in China [70]. However, overdose or long-term usage of Alismatis Rhizoma can cause nephrotoxicity. Yu et al. employed a LC–MS based metabolomic approach to investigate the nephrotoxicity of Alismatis Rhizoma in rats. The results indicated that significant changes in thirteen metabolite biomarkers were detected in the urine after treatment of Alismatis Rhizoma. The metabolomic method could discriminate the extract treated rats from the control rats on days 60, 120, and 180 after treatment. While serious organic renal damage was not observed on histopathology until day 180. The results indicated that LC–MS based metabolomic analysis is an useful tool for predicting the chronic nephrotoxicity induced by Alismatis Rhizoma [71].

### Metabolomics in cardiotoxicity evaluation of TCM

Periplocin, a digitalis-like cardiac glycoside from Periplocae Cortex, has been used widely in clinic for its cardiotonic, anti-inflammatory and anti-tumor effects [72]. To evaluate the cardiotoxicity of periplocin, Li et al. reported an UPLC Q/TOF MS method to reveal the metabolic profiles on neonatal rat cardiomyocytes exposed to periplocin. Eleven biomarkers associated with cardiotoxicity including carnitine, acetylcarnitine, lysoPC, proline, glutamic acid, pyroglutamic acid, leucine, pantothenic acid, tryptophan, indoleacrylic acid and citric acid were identified. The metabolic pathway analysis indicated that these metabolites were associated with amino acid metabolism, energy metabolism and sphingolipid metabolism, which contributes to the cardiotoxicity mechanism of periplocin [73].

Herbal medicines derived from *Aconitum* species, including Aconiti kusnezoffii Radix, Aconiti Radix and Aconiti Lateralis Preparata Radix have a long history of clinical use. These herbs have been shown to exhibit biological effects on various diseases, including rheumatic fever, painful joints, bronchial asthma, gastroenteritis, collapse, syncope, diarrhea, edema and tumors. Modern research revealed that *Aconitum* herbs have potent toxicity, and *Aconitum* alkaloids are not only the active ingredients but also toxic components [74]. Aconitine, mesaconitine, and hypaconitine are the main *Aconitum* alkaloids derived from Aconiti lateralis Radix praeparata, the lateral root of *Aconitum carmichaelii* Debx. These alkaloids have analgesic, antipyretic, and local anesthetic activities and have beneficial effects against rheumatosis and rheumatoid arthritis. But the strong toxicity and the narrow margin between therapeutic and toxic doses limited clinical application of the *Aconitum* alkaloids. Sun et al. investigated the metabolic changes in rats caused by the aconitine, mesaconitine, and hypaconitine using ^1^H NMR and GC–MS. Compared with control group, the results revealed larger deviations in the aconitine and mesaconitine groups and smaller deviations in hypaconitine group, illustrating the different toxicity mechanisms of these alkaloids. Metabolomic analysis indicated that most of the metabolic biomarkers were related to tricarboxylic acid cycle [75].

Aconiti kusnezoffii Radix, the root of *Aconitum kusnezoffii* Reichb., was reported to induce toxicity to heart and central nervous system [76]. Recently, Yan et al. proposed a UPLC Q/TOF MS based metabolomic approach to characterize the phenotypically biochemical perturbations and potential mechanisms of Aconiti kusnezoffii Radix-induced toxicity. The urinary metabolomics revealed serious toxicity to heart and liver. Thirteen metabolites were identified and validated as phenotypic toxicity biomarkers of Aconiti kusnezoffii Radix. These biomarkers were responsible for pentose and glucuronate interconversions, tryptophan metabolism, amino sugar and nucleotide sugar metabolism, taurine and hypotaurine metabolism, ascorbate and aldarate metabolism, fructose and mannose metabolism, and starch and sucrose metabolism [77].

The potential cardiotoxicity of Aconiti Radix (the mother roots of *Aconitum carmichaelii* Debx) was frequently reported because of its narrow therapeutic window. A metabolomic method was performed to characterize the potential mechanisms of Aconiti Radix-induced cardiotoxicity by UPLC Q/TOF MS. Seventeen biomarkers were identified in urinary samples, which were associated with pentose and glucuronate interconversions, alanine, aspartate, and glutamate metabolism [78]. Meanwhile, the levels of the identified toxicity biomarkers were modulated to the normal ranges by Glyeyrrhizae Radix, Paeoniae Alba Radix and Zingiberis Rhizoma. The results indicated that these three compatible herbal medicines could be the effective detoxifying substances against the toxicity of Aconiti Radix [79].

Aconiti Lateralis Radix Praeparata, the lateral or daughter root of *Aconitum carmichaelii* Debx, has a potential cardiotoxicity with a relatively narrow margin of safety. Wang et al. reported a LC–MS metabolomic approach to investigate and compare the metabolic changing of Aconiti Lateralis Radix Praeparata, Aconiti Radix and the processed products. The data demonstrated that both Aconiti Lateralis Radix Praeparata and Aconiti Radix could lead to serious cardiotoxicity in a time- and dose-dependent manner. Sphingolipid metabolism, aminoacyl-tRNA biosynthesis and tryptophan metabolism mainly contributed to the toxicity of Aconiti Lateralis Radix Praeparata and Aconiti Radix [80]. Cai et al. further employed a lipidomics strategy to explore the cardiotoxic mechanisms of Aconiti Lateralis Radix Praeparata and find out potential tissue-specific biomarkers by HPLC Q/TOF MS. Fourteen lipid metabolites, which are primarily involved in phospholipid metabolism, sphingolipid metabolism, saturated fatty acid oxidation and unsaturated fatty acid peroxidation, were identified and considered as the potential biomarkers of the cardiotoxicity induced by Aconiti Lateralis Radix Praeparata [81].

Pinelliae Rhizoma, the dried tuber of *Pinellia ternata* (Thunb.) Breit., is commonly used for treatment of cough, vomiting, infection and inflammation [82]. Zhang et al. proposed a UPLC Q/TOF MS metabolomic approach to elucidate the toxicity of Pinelliae Rhizoma extract in rats. The results indicated that oral administration of Pinelliae Rhizoma did not induce obvious liver and kidney toxicity, but caused certain cardiotoxicity. The identified seven endogenous metabolites indicated the perturbations of phospholipid metabolism, amino acid metabolism and carnitine metabolism in Pinelliae Rhizoma treated rats [83]. According to the TCM theory, processing can reduce the toxicity of Pinelliae Rhizoma. Using the metabolomic approach, Su et al. investigated the mechanisms of raw Pinelliae Rhizoma induced toxicity and toxicity-reducing effect of processing. Consistent with the above report, the metabolomic results also indicated that raw Pinelliae Rhizoma could cause cardiotoxicity. Inhibition of mTOR signaling and activation of the TGF-β pathway contributed to raw Pinelliae Rhizoma-induced cardiotoxicity, and free radical scavenging might be responsible for the toxicity-reducing effect of processing [84].

### Metabolomics in other toxicity evaluation of TCM

In addition to the above-mentioned hepatotoxicity, nephrotoxicity and cardiotoxicity, reproduction toxicity of triptolide is also the main obstacle for its clinical applications. Ma et al. developed a GC–MS based metabolomic approach to evaluate the mechanism of triptolide-induced reproductive toxicity in male mice and identify potential biomarkers for the early detection of spermatogenesis dysfunction. The results indicated that the testicular toxicity of triptolide may be caused by abnormal lipid and energy metabolism in testis via down-regulation of peroxisome proliferator-activated receptor mediated [85].

Cinnabar, a traditional mineral medicine containing more than 96% mercuric sulfide, has been used as a sedative and soporific for more than 2000 years. It was reported that cinnabar can impact central nervous system and cause neurotoxicity through blood–brain barrier [86]. Wei et al. investigated the neurotoxicity of cinnabar in rats by ^1^H NMR based metabolomics combined with multivariate pattern recognition. The metabolite variations induced by cinnabar were characterized by increased levels of glutamate, glutamine, myo-inositol, and choline, as well as decreased levels of γ-amino-*n*-butyrate, taurine, *N*-acetylaspartate and *N*-acetylaspartylglutamate in tissue extracts of the cerebellum and cerebrum. The results indicated that cinnabar induced glutamate excitotoxicity, neuronal cell loss, osmotic state changes, membrane fluidity disruption, and oxidative injury in the brain [87].

Kunsui Radix, the dried root of *Euphorbia kansui* T. N. Liou ex T. P. Wang, was widely used for treatment of edema, ascites, and asthma [88]. The clinical application of Kunsui Radix is greatly restricted since it can induce toxic symptoms such as stomachache, diarrhea, dehydration and respiratory failure. The metabolites responsible for the toxicity of Kunsui Radix were evaluated by ^1^H NMR based metabolomics. The toxicity of Kunsui Radix accumulated with dosing time, and persisted even when treatment was stopped. The metabolomic results revealed that the levels of alanine, lactate, taurine, betaine, hippurate, phenylalanine and glucose were increased, while the levels of succinate, citrate, glycine, creatine and creatinine were decreased. The corresponding biochemical pathways alterations included inhibited tricarboxylic acid cycle, increased anaerobic glycolysis, and perturbed amino acids metabolism [89].

Coptidis Rhizome has been used as a heat-clearing and detoxifying agent in China for 2000 years. Coptidis Rhizome is relatively safe in normal dosage, but an extensive dosage can cause side effects such as diarrhea. A combination of ^1^H NMR and GC–MS based metabolomic approach was applied to discover the endogenous metabolites which related to the diarrheal induced by Coptidis Rhizome. In the study, twelve marker metabolites from ^1^H NMR and eight from GC–MS were identified, among those metabolites, hippurate, acetate, alanine, glycine and glutamate were likely to break the balance of gut microbiota, whereas, lactate and 2-ketoisovalerate were associated with energy metabolism [90].

TCM is generally used in the form of prescriptions (the combination of several different herbal medicines). The bioactive constituents and fundamental mechanisms of most TCM prescriptions remain unclear due to the complex components of remedies. Metabolomics could provide a holistic view and deeper insight into the efficacy and toxicity of TCM prescriptions. It might also be a promising approach to investigate the detoxification of Chinese medicines and reasonable combination of TCM prescriptions. Niuhuang Jiedu Tablet, composed of Realgar, Bovis Calculus Artificialis, Borneolum Synthcticum, Gypsum Fibrosum, Rhei Radix et Rhizoma, Scutellariae Radix, Platycodonis Radix and Glycyrrhizae Radix et Rhizoma, is an effective TCM prescription used for treatment of acute tonsillitis, pharyngitis, periodontitis and mouth ulcer [91]. In the prescription, significant level of realgar is a potentially toxic element. Xu et al. proposed a ^1^H NMR based metabolomic approach to investigate the toxicity of realgar after being counterbalanced by other herbal medicines in Niuhuang Jiedu Tablet. The results showed that it was more secure and much less toxic for counterbalanced realgar in Niuhuang Jiedu Tablet. The effective material bases of toxicity alleviation to realgar were Rhei Radix et Rhizoma, Scutellariae Radix, Platycodonis Radix and Glycyrrhizae Radix et Rhizoma, which regulated energy metabolism, choline metabolism, amino acid metabolism and gut flora disorder affected by realgar exposure [92].

Zhusha Anshen Wan, composed of cinnabar, Coptidis Rhizoma, Angelicae Sinensis Radix, Rehmanniae Radix, Glycyrrhizae Radix et Rhizoma, is a widely used TCM prescription for sedative therapy. Cinnabar is the chief component of Zhusha Anshen Wan and possesses certain toxicity. A metabolomic analysis suggested that Zhusha Anshen Wan may be more secure and much less toxic than cinnabar alone, and the four combined herbal medicines of Zhusha Anshen Wan had the effects of protecting from the toxicity induced by cinnabar alone [93].

Shuanghuanglian injection, composed of Lonicerae japonicae Flos, Scutellariae Radix and Forsythiae Fructus suspensa, is a commonly used TCM preparation with known antimicrobial properties [94]. It was reported that the adverse drug reactions of Shuanghuanglian injection occurred in approximately 2.22–2.56% after clinical exposure and the main adverse drug reactions were hypersensitive response, hemolytic anemia, haematuria and jaundice. The toxicological effects of Shuanghuanglian injection after intravenous administration in Beagle dogs were investigated by a ^1^H NMR-based metabolomic approach. The results revealed increases in serum choline, phosphocholine, ketone body and lactate, but decreases in trimethylamine N-oxide, taurine, leucine, valine, glycine and glutamine, and these findings may underlie the toxicity mechanisms of Shuanghuanglian injection [95].

### Conclusions and perspectives

In recent years, metabolomics analysis has increased markedly in efficacy, quality control, action of mechanism, and active components discovery of TCM. Meanwhile, the toxicity of TCM have attracted a wide range of concerns and aroused many toxicity studies on TCM. Nevertheless, there is no standard and objective basis for TCM toxicity evaluation and no standard for safety assessment up to now, which seriously hinders the toxicological researches of TCM. As a systemic approach, metabolomics focuses on the analysis of global metabolites and their functions in the biological system. It allows quantitative measurement of large numbers of low-molecular endogenous metabolites involved in metabolic pathways, and thus reflects fundamental metabolism status of body. The systematic study of metabolomics is in agreement with TCM theory and may be the best approach to fit the holistic concept of TCM. Therefore, metabolomic analysis is a suitable tool to systematically evaluate toxicity, find potential biomarkers and explore the toxicological mechanisms of TCM.

Despite its potential and advantages, there are still great challenges for the metabolomic applications on toxicology of TCM. Firstly, high sensitivity of metabolites to various genetic and environmental factors might lead to difficult interpretation of data analysis. Secondly, there are still substantial shortcomings for the existing metabolomic techniques to analyze the full spectrum of metabolites. Thirdly, it is difficult to establish relationships between metabolomic results with genomics, proteomics and clinical data. Although there are still many challenges for the development of metabolomics in toxicity evaluation and it is a long way to get it into clinical application, we believe that the comprehensive metabolomic approach is a potentially powerful tool to evaluate toxicology and explore toxicological mechanisms of TCM. It is expectable that with the development of various analytical techniques, metabolomics will play an increasingly critical role in TCM toxicology research and be beneficial to the modernization of TCM.

## Abbreviations

TCM: traditional Chinese medicines
^1^H NMR: proton nuclear magnetic resonance spectroscopy
MS: mass spectrometry
GC: gas chromatography
GC–MS: gas chromatography–mass spectrometry
LC: liquid chromatography
LC–MS: liquid chromatography–mass spectrometry
CE: capillary electrophoresis
CE–MS: capillary electrophoresis–mass spectrometry
UPLC: ultra-performance liquid chromatography
UPLC–MS: ultra-performance liquid chromatography–mass spectrometry
PCA: principal component analysis
HCA: hierarchical cluster analysis
PLS-DA: partial least squares discriminant analysis
OPLS-DA: orthogonal partial least squares discriminant analysis
HMDB: human metabolome database
KEGG: Kyoto Encyclopedia of Genes and Genomes
BiGG: biochemical genetic and genomic
HPLC Q/TOF MS: high performance liquid chromatography coupled with quadrupole time-of-flight mass spectrometry
UPLC Q/TOF MS: ultra-performance liquid chromatography coupled with quadrupole time-of-flight mass spectrometry
